# Expression of hypoxia-related markers in inflammatory myofibroblastic tumors of the head and neck

**DOI:** 10.1186/1477-7819-11-294

**Published:** 2013-11-19

**Authors:** Kui-Rong Wang, Tao Jiang, Ting-Ting Wu, Shui-Hong Zhou, Hong-Tian Yao, Qin-Ying Wang, Zhong-Jie Lu

**Affiliations:** 1 Department of Anaesthesiology, The First Affiliated Hospital, College of Medicine, Zhejiang University, Hangzhou 310003, China; 2 Department of Otolaryngology, The First Affiliated Hospital, College of Medicine, Zhejiang University, Hangzhou 310003, China; 3 Current address: Department of Otolaryngology, Yinzhou People’s Hospital of Ningbo City, Zhejiang Province, China; 4 Department of Pathology, The First Affiliated Hospital, College of Medicine, Zhejiang University, Hangzhou 310003, China; 5 Department of Radiotherapy, The First Affiliated Hospital, College of Medicine, Zhejiang University, Hangzhou 310003, China

**Keywords:** Glucose transporter-1, Hypoxia-inducible factor 1α, Inflammatory myofibroblastic tumor, PI3K/Akt pathway, Prognosis, Recurrence

## Abstract

**Background:**

The etiology of inflammatory myofibroblastic tumors (IMTs) is controversial and the prognosis is unpredictable. Previous studies have not investigated the expression of hypoxia-related markers in IMTs.

**Methods:**

Between 2002 and 2012, 12 consecutive patients with histologically proven IMTs were enrolled in the study. Immunohistochemistry was used to detect GLUT-1, HIF-1α, PI3K, and p-Akt expression in paraffin-embedded tumor specimens. Associations among GLUT-1, HIF-1α, PI3K, and p-Akt protein expression and clinical parameters were investigated.

**Results:**

The mean duration of follow-up was 52.1 months (range, 11 to 132 months). Six patients had local recurrence. GLUT-1, HIF-1α, PI3K, and p-Akt expression were detected in 41.7%, 50.0%, 33.3%, and 41.7% of patients, respectively. Fisher’s exact test revealed significant correlations between recurrence of IMT and PI3K expression (*P* = 0.01) and p-Akt expression (*P* = 0.015). Univariate analyses revealed significant correlations between survival and GLUT-1 expression (*P* = 0.028), PI3K expression (*P* = 0.006), and p-Akt expression (*P* = 0.028). Multivariate analysis did not show a significant relationship between survival and GLUT-1, HIF-1α, PI3K, or p-Akt. Spearman rank correlation analysis showed significant correlations between HIF-1α and PI3K expression (*r* = 0.707, *P* = 0.01) and between p-Akt and PI3K expression (*r* = 0.837, *P* = 0.001).

**Conclusions:**

Although our results are inconclusive owing to the small sample size, they suggest that PI3K and p-Akt expression may play a role in the recurrence of IMTs of the head and neck.

## Background

Inflammatory myofibroblastic tumor (IMT) is an intermediated-grade tumor, according to the World Health Organizationclassification, and has a potential for recurrence and rare metastasis [1]. However, the etiology of IMT is controversial, and its prognosis is unpredictable [2]. Previous studies, including ours, have shown that the biological behavior of the lesion is associated with location, cellar atypia, the presence of ganglion-like cells, p53, DNA aneuploidy, and anaplastic lymphoma kinase (ALK) [3,4]. However, evidence that these factors are prognostic markers is inconclusive. A number of studies have found high ^18^ F-2-fluoro-2-deoxy-D-glucose (^18^ F-FDG) uptake in IMTs [5,6]. Increased uptake of FDG, a glucose analog, directly reflects a high glucose metabolic rate in IMTs. Several studies have demonstrated that glucose transporter-1(GLUT-1) plays a significant role in the glucose metabolism of malignant tumors and may contribute to increased FDG uptake [7-9]. GLUT-1 is thought to be an intrinsic marker of hypoxia in some tumors [7-9]; however, the expression of hypoxia-related markers has not been investigated in IMTs.

Hypoxia is a common pathophysiological condition in tumors, caused by the tumor outgrowing its vascular supply [10]. Hypoxia is an independent negative prognostic marker that contributes to cancer progression by affecting the behavior of the cancer cells [11]. Hypoxia-inducible factor 1 (HIF-1) plays an important role during these processes [12]. HIF-1 is composed of two subunits: HIF-1α and HIF-1β. Under normoxia, HIF-1α is degraded by the von Hippel-Lindau-dependent ubiquitin-proteasome pathway [13]. However, it is rapidly stabilized under hypoxic conditions. HIF-1α binds to the hypoxia-response elements in the promoter region of target genes, including GLUT-1, which mediate an increase in oxygen availability and energy supply, and enable metabolic adaptation to hypoxia [14]. GLUT-1 expression increases under hypoxia, which is consistent with findings of increased glucose uptake, increased adaptive changes to glycolytic metabolism, and increased cellular proliferation in cancer cells.

HIF-1α is upregulated in a wide range of solid tumors in human beings, and overexpression of HIF-1α is associated with tumor aggressiveness and poor prognosis [15,16]. Thus, HIF-1α is a novel therapeutic target for several solid tumors [17,18]. However, the mechanisms regulating HIF-1α activity are not well understood. Oncogenic signaling pathways, such as the phosphatidylinositol 3-kinase (PI3K)/protein kinase B (Akt) pathway, may regulate HIF-1α [19]. Moreover, the PI3K/Akt pathway has been shown to promote GLUT-1 cell-surface trafficking and activity [20].

In this study, we used immunohistochemistry to determine the levels of PI3K, p-Akt, HIF-1α, and Glut-1 protein expression in IMTs of the head and neck and assessed the relationship among these proteins.

## Methods

### Patients

The subjects were 12 consecutive patients treated between 2002 and 2012 at The First Affiliated Hospital with histologically proven IMT. Data were obtained from the hospital surgical pathology files. Our study was approved by the Institutional Review Board of The First Affiliated Hospital, College of Medicine, Zhejiang University, and written informed consent was obtained from each patient before inclusion.

### Immunohistochemistry

Immunohistochemistry was performed on formalin-fixed paraffin-embedded tissue blocks prepared from biopsies of the primary lesion of each subject. The biopsy tissue was cut into 4-μm sections and analyzed using an EliVision™ Plus IHC kit (Fuzhou Maixin Biotechnology Development, China). Table 1 shows the primary antibody, source, and dilution used. Briefly, the sections were deparaffinized with xylene and dehydrated through an ethanol series, then antigen retrieval was performed with a microwave oven over two 10-min cycles. Endogenous peroxidase activity was blocked by incubating the slides in 1.5% hydrogen peroxide in absolute methanol at room temperature for 10 min. Primary antibodies were applied for 1 hour at room temperature, followed by 50 μl of polymer enhancer for 20 min and 50 μl of polymerized horseradish peroxidase-anti-mouse immunoglobulin G (IgG) (DAB Kit; Maixin Biological) for 30 min. The reaction products were visualized using 3,3′ diaminobenzidine (DAB Kit; Maixin Biological), and the sections were counterstained with hematoxylin and eosin, dehydrated, and examined under a light microscope. Tris-buffered saline was used in place of the primary antibody for negative controls. Erythrocytes, which were present in all sections, served as internal controls for GLUT-1 to ensure constant immunostaining intensity.

**Table 1:** Immunohistochemical molecular markers

**Markers**	**Source**	**Dilution**
GLUT-1	Santa Cruz Biotechnology	1:50
HIF-1α	Santa Cruz Biotechnology	1:100
PI3K	Santa Cruz Biotechnology	1:100
p-Akt	Santa Cruz Biotechnology	1:100
ALK	Dako, Carpinteria, CA, USA	1:100
Ki-67	Santa Cruz Biotechnology	1:100
Vimentin	Dako, Carpinteria, CA, USA	1:100
S-100 protein	Dako, Carpinteria, CA, USA	1:400
Desmin	Dako, Carpinteria, CA, USA	1:100
α-SMA	Dako, Carpinteria, CA, USA	1:5000
CD68	Dako, Carpinteria, CA, USA	1:300
CD34	Dako, Carpinteria, CA, USA	1:50

ALK, anaplastic lymphoma kinase; α-SMA, α-smooth muscle actin; GLUT-1, glucose transporter-1; HIF, hypoxia-inducible factor.

GLUT-1, HIF-1α, PI3K, and p-Akt levels were evaluated by the same investigator (H-TY), who was blinded to the clinical and follow-up data. GLUT-1 expression was considered positive only if distinct membrane staining was present. HIF-1α, PI3K, and p-AKT proteins were observed in the nucleus and cytoplasm. Protein analysis was performed in ten random high-power fields within each of which 100 tumor cells were counted for each case and for all antibodies. The percentage of positive cells was calculated by dividing the number of positive tumor cells by the total number of tumor cells counted. A sample was considered negative if <25% of the cells were stained.

### Follow-up

The patients were scheduled for follow-up visits every 6 months after the initial surgery. Follow-up consisted of a routine physical examination and computed tomography (CT) or magnetic resonance imaging (MRI) of the primary site. A combined ^18^ F-FDG positronemission tomography (PET) and CT scan was performed on one patient. Patient follow-up was reported up to the date last seen in the clinic.

### Statistical analysis

The Statistical Package for the Social Sciences version 19 for Windows (SPSS Inc., Chicago, IL, USA) was used to conduct the statistical tests. Associations among GLUT-1, HIF-1α, PI3K, and p-Akt protein expression and pretreatment clinical parameters were analyzed using the chi-squared and Fisher’s exact tests. A *P *value <0.05 was deemed to indicate statistical significance. Correlation analysis was performed using Spearman’s rank correlation.

## Results and discussion

### Patients’ characteristics

The clinicopathological findings (age, sex, tumor site, recurrence, metastasis, and follow-up) are shown in Table 2. The subjects included four men and eight women with a mean age of 44.4 years (range, 22 to 64 years). Tumors were located in the maxillary sinus (*n* = 4, 33.3%), tonsils (*n* = 2, 16.7%), larynx (*n* = 1, 8.3%), hypopharynx (*n* = 1, 8.3%), maxillare (*n* = 1, 8.3%), tongue (*n* = 1, 8.3%), neck (*n* = 1, 8.3%), and floor of the mouth (*n* = 1, 8.3%). No fever, weight loss, malaise, or laboratory abnormality (anemia, thrombocytosis, elevated erythrocyte sedimentation rate) was observed in any patient.

**Table 2:** **Clinicopathological findings and follow**-**up for the 12 studied cases of inflammatory myofibroblastic tumor**

**Case**	**Sex**	**Age**	**Site**	**CT, MRI, PET**	**Treatment**	**Recurrence**	**Metastasis**	**Follow**-**up**
1	M	64	Larynx	/	Laryngeal fissure, complete excision, clear margin	No	No	No evidence of disease(68 months)
2	M	22	Tonsil	/	Tonsillectomy, clear margin	No	No	No evidence of disease(84 months)
3	M	33	Maxillare	CT showed a softtissue mass in the right maxillary alveolar bone. The local bone of maxillary alveolar and inferior wall of right maxillary sinus, and the mass extended into the right maxillary sinus, mild to moderate enhancement on contrast-enhancement.	Local excision	Yes, 6 years after first surgery. Total maxillectomy was performed.	No	No evidence of disease(16 months after second surgery)
4	M	48	Tongue	CT showed a 3.7 × 1.7 cm irregular softtissue mass in the left base of tongue, strong enhancement on contrast-enhanced imaging.	Local complete excision, clear margin	No	No	No evidence of disease(41 months)
5	F	61	Tonsil	CT showed a 2.6 × 1.8 cm irregular soft tissue mass between the left tonsil and the base of the tongue with no enhancement on contrast-enhanced imaging.	Left tonsillectomy and mass excision, clear margin	No	No	No evidence of disease(23 months)
6	F	46	Hypopharynx	MRI showed that a 1.3 × 2.2 cm mass in the right pyriform sinus. Isointense and slight hypointense on T1-weighted imaging, hyperintense on T2 -weighted imaging, heterogeneous enhancement on contrast-enhanced T1-weighted MRI images.	Excision under suspension laryngoscopy	Yes, 37 months after initial surgery. Second surgery was performed via lateral neck incision.	No	No evidence of disease(10 months after second surgery)
7	F	46	Maxillary sinus	CT showed a diffuse softtissue massin the right maxillary sinus with destruction of the maxillary bone anteriorly and inwardly that extended into the orbit causing exophthalmos anteriorly.	Exploratory operation, oral corticosteroids	Yes, 2 months after initial treatment. Then the patient received total maxillectomy and exenteration + radiotherapy postoperation.1 month after second surgery, recurrence occurred.	Metastasis to cervical lymph node	Died of disease (13 months after initial treatment)
8	F	25	Mouth floor	/	Local excision, clear margin	No	No	No evidence of disease(132 months)
9	F	63	Maxillary sinus	A homogeneous mass in the left maxillary sinus on the CT scan.	Partial maxillectomy neck dissection, 50 Gy radiotherapy was performed.	8 months after initial treatment, occurrence was occurred. Another 50 Gy radiotherapy was given.	Metastasis to cervical lymph node	Died of disease (11 months after first treatment)
10	F	48	Maxillary sinus	CT showed a softtissue expansile mass in the left maxillary sinus.	Partial maxillectomy + corticosteroids	Recurrence 6 months after initial treatment. Total maxillectomy + corticosteroids.	No	No evidence of disease(70 months after first treatment)
11	F	34	Maxillary sinus	CT showed a softtissue expansile mass in the left maxillary sinus extending to the nasal septum	Caldwell-Luc operation	Recurrence 1 month after first surgery. Partial maxillectomy + oral prednisone. Tumor was not contained.3 months later, 60Gy radiotherapy also did not contain the tumor. Endoscopic surgery and total maxillectomy did not control the tumor.	No	Died of disease (35 months after initial treatment)
				Follow-up showed PET/CT showed high FDG uptake in the left maxillary sinus.				
12	F	43	Neck	A 3 × 4 × 11 cm well-defined mass in the right neck, hyperintense on T1,hypointense on T2, heterogeneous enhancement on contrast-enhanced T1-weighted MRI images. MRI findings suggested neurogenic tumor. CT showed a well-defined, heterogeneous mass in the right neck, mild enhancementon contrast-enhanced imaging.	Complete excision, clear margin	No	No	No evidence of disease(13 months)

**Figure 1:**
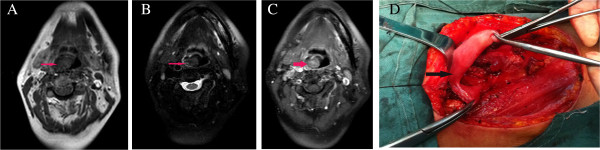
**Magnetic resonance imaging revealed a 1.3 × 2.2-cm mass in the right pyriform sinus. (A)** Signals were isointense and slightly hypointense on the T1-weighted imaging, **(B)** hyperintense on T2-weighted imaging, and **(C)** heterogeneous enhancement was observed on the contrast-enhanced T1-weighted MRI images. **(D)** The patient underwent complete resection via a lateral neck incision and achieved a clear margin at second surgery.

**Figure 2:**
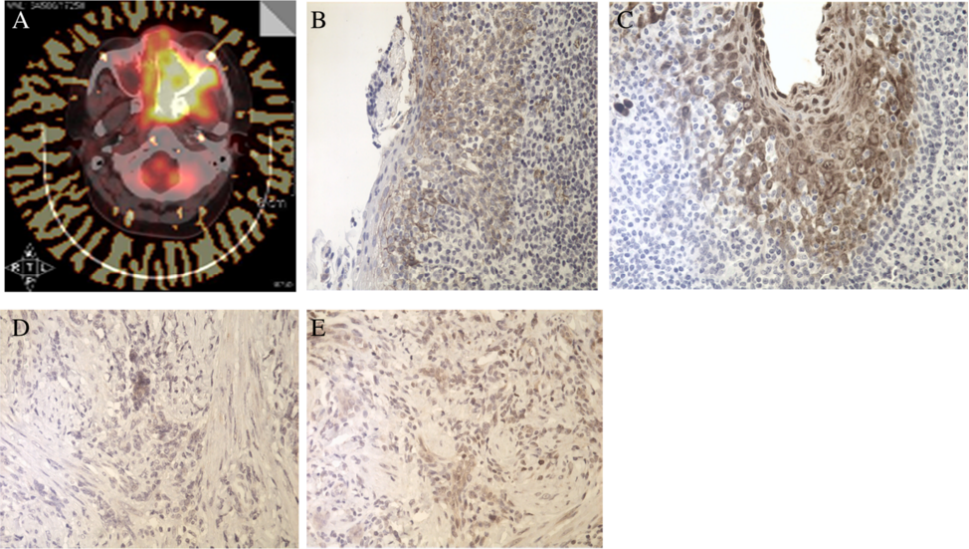
**Positronemission tomography**/**computed tomography. (A)** High 2-fluoro-2-deoxy-D-glucoseuptake in the left maxillary sinus. Expression of **(B)** GLUT-1, **(C)** HIF-1α, **(D)** PI3Kand **(E)** p-Aktwas positive.

Available IMT radiological images varied. CT, MRI, or FDG-PET/CT scans were available for nine patients. CT images were available for eight patients and showed homogenous (one case) or heterogeneous (seven cases) softtissue masses. Contrast-enhanced CT images were available for four patients: one case of IMT of the tonsil showed no enhancement, mild enhancement was found in one case of hypopharynx IMT, mild to moderate enhancement was detected in one of the maxillary sinus cases, and the IMT of the tongue showed strong enhancement. MRI data were available for two patients. In one patient, the T1-weighted signals were isointense and slightly hypointense and the T2-weighted signals were hyperintense. In the other patient, the T1 images were hyperintense and the T2 images were hypointense. The contrast-enhanced T1-weighted MRI images showed heterogeneous enhancement in both patients (Figure 1). FDG-PET/CT revealed FDG uptake in the left maxillary sinus (Figure 2A).

Surgery was the initial treatment for all patients. Six patients underwent complete excision to achieve a clear margin. Local excision was performed on five patients with IMT of the maxillary sinus (*n* = 5) and maxillare (*n* = 1). Of those, two patients received corticosteroids following surgery, one patient received postoperative radiotherapy, and two patients receive no postoperative adjuvant treatment. The IMT of the larynx was excised using suspension laryngoscopy.

Follow-up ranged from 11 to 132 months (mean, 52.1 months). Six patients experienced local recurrence. These patients had received local excision as the initial treatment, indicating that local excision cannot guarantee negative resection margins. Two of the patients who experienced a local recurrence had cervical metastasis. Five of the six patients underwent a second surgery. Of those, three received total maxillectomy, and two of these three received postoperative chemotherapy or radiotherapy. One patient received a partial maxillectomy, and one patient with a hypopharyngeal IMT underwent complete resection via a lateral neck incision and achieved a clear margin. One patient refused a second surgery, but accepted postoperative radiotherapy (50 Gy). Of these six patients, three with IMT of the maxillary sinus died of uncontrolled disease, including the two patients with metastatic cervical lymph nodes. No evidence of local recurrence was found in the clear margin of the six patients at the initial surgery. Furthermore, no local recurrence was found in the three patients who had clear margins following additional surgery. Nine patients were alive at the time of the last follow-up.

### Pathological findings

Microscopically, the tumor is composed of spindle cells with various inflammatory cells, such as plasma cells, lymphocytes, and occasional eosinophils. The compact spindle cell pattern is characterized by a cellular proliferation of spindle cells with a fascicular or storiform architecture in a collagenous stroma. Nuclear atypia, frequent mitoses, and atypical mitotic figures are infrequent in some cases.

### Immunohistochemical findings

All cases were immunohistochemically positive for vimentin staining. The positive expression of α-smooth muscle actin (α-SMA), desmin, and ALK was 67.7% (8/12), 25.0% (3/12), and 33.3% (4/12), respectively. S-100, CD34, and CD68 were negative in the tumor cells in all cases. Ki-67 expression was low (<5%) in all cases.

The rates of GLUT-1, HIF-1α, PI3K, and p-Akt expression were 41.7% (5/12), 50.0% (6/12), 33.3 (4/12), and 41.7% (5/12), respectively (Table 3, Figure 2B-E). Of the six patients who experienced recurrence, four (67.7%) were positive for GLUT-1, five (83.3%) were positive for HIF-1α, four (67.7%) were positive for PI3K, and five (83.3%) were positive for p-Akt. Fisher’s exact test revealed significant correlations between lesion recurrence and the expression of PI3K (*P* = 0.01) and p-Akt (*P* = 0.015); however, no significant correlation was found between recurrence and the ex pression of GLUT-1 (*P* = 0.242) or HIF-1α (*P* = 0.08). The expression of GLUT-1, HIF-1α, PI3K, and p-Akt was positive in the two patients with cervical lymph node metastasis, in the three patients who died, and in the patient who underwent PET/CT during follow-up.

**Table 3:** **Results of Glut**-**1**, **HIF**-**1α**, **PI3K**, **and p**-**Akt and outcome in 12 inflammatory myofibroblastic tumors of head and neck**

**Case**	**Site**	**Recurrence**	**Metastasis**	**Follow**-**up**	**Expression of**			
					**Glut**-**1**	**HIF**-**1α**	**PI3K**	**p**-**Akt**
1	Larynx	No	No	No evidence of disease (68 months)	−	+	−	−
2	Tonsil	No	No	No evidence of disease (84 months)	+	−	−	−
3	Maxillare	Yes	No	No evidence of disease (88 months after first surgery)	−	−	−	+
4	Tongue	No	No	No evidence of disease (41 months)	−	−	−	−
5	Tonsil	No	No	No evidence of disease (23 months)	−	−	−	−
6	Hypopharynx	Yes	No	No evidence of disease (47 months after first surgery)	−	+	+	+
7	Maxillary sinus	Yes	Metastasis to cervical lymph node	Died of disease (13 months after initial treatment)	+	+	+	+
8	Mouth floor	No	No	No evidence of disease (132 months)	−	−	−	−
9	Maxillary sinus	Yes	Metastasis to cervical lymph node	Died of disease (11 months after first treatment)	+	+	+	+
10	Maxillary sinus	Yes	No	No evidence of disease (70 months after first treatment)	+	+	−	−
11	Maxillary sinus	Yes	No	Died of disease (35 months after initial treatment)	+	+	+	+
12	Neck	No	No	No evidence of disease (13 months)	−	−	−	−

+, positive; −, negative.

Mean overall survival was 102 months (95% confidence interval, 73 to 131) in our sample. The 5-year overall survival probability was 72.9%. Univariate analyses revealed a significant correlation between survival and GLUT-1 (*P* = 0.028), PI3K (*P* = 0.006), and p-Akt (*P* = 0.028) expression. The multivariate analysis revealed no statistically significant relationship between survival and levels of GLUT-1, HIF-1α, PI3K, and p-Akt.

Spearman’s rank correlation analysis revealed a significant correlation between HIF-1α and PI3K expression (*r* = 0.707, *P* = 0.01) and between p-Akt and PI3K expression (*r* = 0.837, *P* = 0.001). No correlation was found between the expression of GLUT-1 and HIF-1α, PI3K, or p-Akt expression (*P* > 0.05).

IMTs of the head and neck are relatively rare [3], and most previous reports have been case studies. Two relatively large studies of IMTs of the head and neck conducted by Ong *et al*. [2] in 28 patients and by Chen *et al*. [21] in 10 patients reported a high recurrence rate. Our findings are consistent with those series. Our recurrence rate was 50% (6/12), generally within 1 year after the initial treatment, and the mean time to recurrence was 21 months (range, 1 to 72 months). The average follow-up period was 52.1 months (range, 11 to 132 months). Two patients (16.7%) developed cervical lymph node metastases, three (25.0%) died as a result of the IMT, and nine patients were alive at the time of the last follow-up.

The clinical findings and pathological features that predict the course of IMTs are poorly understood. In our series, three patients with tumors in the maxillary sinus died. Local excision had been performed in all of the patients who experienced recurrence. Our results are similar to those of Jiang *et al*. [3], who reported significant correlations between histological atypicality and recurrence and metastasis. Ong and colleagues [2] reported that tumor location, depth, cytology pattern, pseudocapsule component, and mitotic figure did not significantly affect time to relapse and overall survival. They argued that surgical margin was the most significant and independent predictor of local relapse [2]. However, other investigators have reported that IMT location is associated with recurrence [2-4]. Coffin *et al*. [4] reported that the recurrence of IMTs in the abdominopelvic area was higher than that in other locations. Ong and colleagues [2] suggested that the poor outcome associated with IMTs of the maxillary sinus and nasal cavity stemmed from the fact that lesions in these locations could readily extend superiorly to Ohngren’s line and involve the orbit and skull base. They hypothesized that involvement of these vital organs restricted the surgical resection, resulting in positive surgical margins. Although our sample size was small, our findings support this hypothesis.

The results of previous investigations of possible morphological and genetic prognostic indicators for IMTs were inconclusive [2-4]. Ong *et al*. [2] found that immunohistochemical biomarkers were not significantly correlated with recurrence and prognosis. Coffin *et al*. [4] and Jiang *et al*. [3] found that ALK reactivity was a favorable prognostic indicator for IMT. We investigated molecular biomarkers associated with IMTs of the head and neck using immunohistochemistry to assess glucose metabolism in the lesions. In this, the protein expression rates were 41.7% for GLUT-1, 50.0% for HIF-1α, 33.3% for PI3K, and 41.7% for p-Akt, and the recurrence of IMT was significantly associated with PI3K and p-Akt expression. Although the expression of GLUT-1 and HIF-1α was not significantly associated with recurrence, 67.7% (4/6) of the patients were positive for GLUT-1 and 83.3% (5/6) were positive for HIF-1α. The expression of GLUT-1, HIF-1α, PI3K, and p-Akt was positive in the two patients who developed cervical lymph node metastasis, the three patients who died from the IMT, and the patient who underwent PET and CT during follow-up. In a previous study, we found that GLUT-1, HIF-1, PI3K, and p-Akt were expressed in a case of ceruminous adenoma [22]. Furthermore, we found that the rate of GLUT-1, PI3K, and p-Akt protein in patients with adenoid cystic cancerof the head and neck was 38.1%, 38.1%, and 50.0%, respectively [23].

In this series, the 5-year overall survival probability was 72.9%. Univariate analysis revealed a significant correlation between survival and the expression of GLUT-1, PI3K, and p-Akt. Multivariable analysis revealed no significant relationship between survival and GLUT-1, HIF-1α, PI3K, or p-Akt levels. In a previous study, we found a significant correlation between GLUT-1 and HIF-1α (*r* = 0.504; *P* = 0.000) expression and suggested that increased GLUT-1 expression was an independent predictor of survival for laryngeal carcinoma [12]. Moreover, in cases of adenoid cystic cancer of the head and neck we found significant correlations between the expression of GLUT-1 and PI3K, GLUT-1 and p-Akt, and p-Akt and PI3K [23]. One possible explanation is that the PI3K/Akt signal-transduction cascade regulates HIF-1α expression [24], which can activate the transcription of more than simply its target genes [25], including GLUT-1. Alternatively, GLUT-1 expression may be directly activated by the PI3K/Akt pathway, which has been shown to promote GLUT-1 cell-surface trafficking and activity [20]. However, in this study, we found significant correlations only between HIF-1α and PI3K, and between p-Akt and PI3K expression. No correlation was found between GLUT-1 expression and that of HIF-1α, PI3K, or p-Akt. The absence of a significant correlation between HIF-1α and proteins of PI3K/Akt pathway may be attributable to our small sample size. Thus, further studies of the relationship between GLUT-1 expression and HIF-1α and proteins of PI3K/Akt pathway in IMTs of the head and neck are warranted.

## Conclusions

Although our results are inconclusive, owing to small sample size, we suggest that expression of PI3K and p-Akt may play a role in the recurrence of IMTs of the head and neck. Univariate analyses revealed significant correlations between GLUT-1, PI3K, and p-Akt expression and survival. However, multivariate analysis did not find statistically significant relationships between survival and GLUT-1, HIF-1α, PI3K, p-Akt expression. Further study is needed to confirm our findings.

## Abbreviations

ALK: Anaplastic lymphoma kinase; CT: Computed tomography; 18 F-FDG: ^18^ F-2-fluoro-2-deoxy-D-glucose; GLUT-1: Glucose transporter-1; HIF: Hypoxia-inducible factor; IMT: Inflammatory myofibroblastic tumor; MRI: Magnetic resonance imaging; PI3K/Akt: Phosphatidylinositol 3-kinase/protein kinase B pathway; PET: Positron emission tomography; α-SMA: α-smooth muscle actin.

## Competing interests

The authors declare that they have no competing interests.

## Authors’ contributions

S-HZ conceived and designed the study, performed experiments, participated in data collection, analyzed the data, and drafted the manuscript. H-TY contributed to the study design and performed the immunohistochemistry. K-RW participated in study design and aided surgeries. TJ ,T-TW, Q-YW, and Z-JL collected the materials and follow-up data. All authors read and approved the final manuscript.
